# Increased adolescent game usage and health-related risk behaviors during the COVID-19 pandemic

**DOI:** 10.1007/s12144-023-04466-8

**Published:** 2023-04-03

**Authors:** Young-Jae Kim, Chan Sol Lee, Seung-Woo Kang

**Affiliations:** Department of Physical Education of Chung, Ang University, 06974 Seoul, Republic of Korea

**Keywords:** Gaming, Adolescent, Health risk, Lockdown, COVID-19, Immersion, Level of participation, Game experience

## Abstract

This study examines adolescent game usage and corresponding health-related risk behaviors during a period of limited social interaction and activity due to the COVID-19 pandemic. Participants included 225 middle- and 225 high-school students in Seoul who completed a survey online from October 1 to 30, 2021. The study measured participants’ game usage level and the health-related risk behavior index. Findings showed that participants who engaged in excessive gaming showed higher levels of health-related risk behaviors. A multivariate analysis of variance was conducted to compare the health-related risk behaviors of students in the general, potential, and high-risk groups on excessive gaming. Results indicated that female students in the high-risk group showed higher stress levels and fatigue (*f* = 5.549, *p* < .01, Cohen’s *d* = 0.016) than the males of the same group. However, male students showed higher physical inactivity levels (*f* = 3.195, *p* > .05, Cohen’s *d* = 0.009) than females. The post hoc test indicated clear sex distinctions among the general, potential, and high-risk groups on excessive gaming (*p* < .001). Among the high-risk game usage group, female students displayed a higher level of risk behaviors than males. Adolescent gaming addiction should be considered an emotional and behavioral disorder for which parental guidance and support are needed, and counseling experts and professionals must come together to provide a cure and reform program.

The COVID-19 pandemic caused unprecedented changes to adolescents’ lives as they were unable to attend school and transitioned from an in-person to online learning environment. These drastic changes to daily life have had adverse effects on their psychological and physical well-being (Alt et al., 2021; Gracia et al., 2021; Park, 2021; Ravens-Sieberer et al., 2021). Mental health issues such as depression and anxiety experienced during the formative years of adolescence can persist into adulthood (Rapee et al., 2019; Trucco et al., 2022). To resolve these negative feelings, young adults have turned to gaming as a leisure activity (Caner & Evgin, 2021; Thomson et al., 2021). However, some studies suggest that stress relief via gaming can positively or negatively amplify any preexisting mental or emotional problems (Koban et al., 2021). Additionally, experiencing a major traumatic event can negatively impact adolescents’ mental well-being (Dick et al., 2021; McLaughlin & Lambert, 2017; Trucco et al., 2022). In other words, the current COVID-19 pandemic can be considered a traumatic event endangering young adults’ physical and mental health as their lives have been disarrayed.

## The impact of gaming on youth

One of the greatest problems affecting the youth is the increase in gaming due to COVID-19. According to the 2020 Game Usage Survey and 2021 Comprehensive Survey on Game Overindulgence, the percentage of children and adolescents (elementary school grades 4–6, middle-, and high-school students) who engage in gaming increased in 2019 (Kang & Kwon, 2021; Korea Creative Content Agency, 2020). Thus, it can be assumed that adolescent health will be negatively impacted owing to social isolation and increased gaming as the COVID-19 pandemic continues. The effects of the pandemic are occurring at a crucial stage in adolescents’ physical, cognitive, and mental development (Trucco et al., 2022). Moreover, adolescent health-related risk behaviors have become apparent as they became unable to use public spaces and engage in physical activity or social interactions due to COVID-19 lockdowns (Mayfield & Fogger, 2022; Roche et al., 2020; Waselewski et al., 2020). As COVID-19-induced social isolation led to a more sedentary lifestyle, teenagers turned to online environments for social interaction, such as online gaming.

### Status of health risk behavior related to gaming

Gaming is perceived as a passionate activity that provides a satisfactory leisure experience (Lalande et al., 2017). Although determining whether gaming is a healthy hobby that alleviates stress or an activity that brings about excessive gaming or addiction is personal, most studies consider gaming in a negative light (Bamps et al., 2022; Kim, 2019). However, gaming has also been found to bring about positive psychological effects such as conflict and anxiety relief, amicable personal relations, self-identity, and self-esteem (Harris et al., 2020; Lee & Kim, 2015; Seo, 2007). Hong Kong faced a surge in adolescent excessive gaming and addiction due to COVID-19; however, loneliness due to social isolation was noted to be the greater problem (Zhu et al., 2021). Thus, it can be said that gaming usage that alleviates loneliness can lower health-related risk behaviors such as suicide.

Nevertheless, studies have focused on the negative impacts of excessive gaming behavior and how to prevent them (Dredge & Chen, 2020). In this way, gaming plays a vital role in adolescents’ development and growth (Oades-Sese et al., 2021; Widmer et al., 1996) as they explore autonomy and construct their self-identity to formulate and pursue their societal goals (Gordon & Caltabiano, 1996; Xie et al., 2020; Kim et al., 2006) asserted that immersive game behavior can bring about mental health issues such as anxiety, depression, and suicidal thoughts and is closely tied with academic stress, unstable personal relations, and familial struggles (Jeong et al., 2019; Kim, 2015). Additionally, Al-Sharqi and Hasan (2022) cited that while there might be slight differences in health-related behaviors among male and female students that game excessively, this activity has a negative impact on the lives of both sexes. Furthermore, Bradshaw et al. (2013) deemed that certain sources of exposure to violence (media, video games, cyberbullying) affected the results of their study. Therefore, a deeper investigation examining how various games affect health-related risk behaviors differently is necessary.

### Health risk behavior by gender

Differences in the types of risk behaviors must be considered when investigating differences in risk behaviors among the sexes (Centers for Disease Control and Prevention, 2010). Students who engage in excessive gaming display the lowest levels of life satisfaction and self-esteem while having the highest levels of depression, anxiety, and academic stress. Thus, the COVID-19 pandemic and consequent social distancing policies increased adolescent gaming, ultimately impacting health-related risk behaviors (Donati et al., 2021).

Our study specifically examines the relationship between excessive gaming and health risks. Dredge and Chen (2020) found that online gaming and social media usage are important in investigating the negative effects of internet use, and that they also affect school life. Various studies have found that adolescents face the most internal conflicts, as they are still unstable but developing physically and mentally at a progressive rate (Hyun et al., 2004); they sometimes seek out activities such as drinking or smoking to alleviate the symptoms of internal conflict (Eaton et al., 2008).

Young adults who show a serious level of excessive gaming and health-related risk behaviors are being impacted during the pandemic (Han et al., 2022; She et al., 2021). However, studies on which health-related risk behaviors are impacted by the severity of game usage are still lacking. Therefore, the present study aims to determine the types of health-related risk behaviors prevalent among adolescents during the social distancing and lockdowns implemented during the COVID-19 pandemic. The following hypotheses were set following previous research on the severity of adolescent game usage and health-related risk behavior.

1. Health-related risk behavior differs among adolescents based on their social background.
2. Health-related risk behavior differs among adolescents based on their game participation level.
3. Game usage levels differ among adolescents based on their sex and school year, and students in higher grade levels engage in more health-related risk behaviors.
4. Health-related risk behavior differs among adolescents depending on the types of games played.

## Method

### Sample and participants

This study enlisted 225 middle-school and 225 high-school students residing in Seoul and Gyeonggi-do, Korea, using the convenience sampling method, a form of non-probability sampling. Participants’ characteristics are shown in Table 1. Teachers used snowball sampling to reach more respondents. Snowball sampling was first conducted for participants who primarily preferred mobile games, and subsequently, for those who preferred online games. In this study, “online games” refer to games played using a computer, and “mobile games” refer to games played using a mobile phone. Once invalid responses were removed, 208 middle-school and 180 high-school students remained. This study was conducted online from October 1 to October 30, 2021. The teacher in charge explained the survey process, and the Google online link was provided to the students. Then, they were instructed to proceed with the survey after returning to their homes if their parents’ consent was obtained.

**Table 1:** Participant characteristics

Variable		*n*(%)
Sex	Male	212(54.6)
	Female	176(45.4)
	Total	388
School level	Middle School	208(53.6)
	High School	180(46.4)
	Total	388
Type of games played	Online Games	216(49.1)
	Mobile Games	172(39.1)
	Total	388
Hours per day spent gaming	> 30 min-1 h	38(9.8)
	< 2 h	54(13.9)
	2–3 h	140(36.1)
	> 3 h	156(40.2)
	Total	388
Gaming experience	< 1 Year	58(14.9)
	1 ~ 2 Years	73(18.8)
	3 ~ 4 Years	141(36.3)
	> 5 Years	116(29.9)
	Total	388
Game usage level	General-User Group	206(53.1)
	Potential-Risk Group	60(15.5)
	High-Risk Group	122(31.4)
	Total	388

During that time in Korea, a low-level lockdown was in place. Participants were recruited through a Google survey, and answered a self-administered questionnaire. Of the 450 surveys, 62 were excluded due to duplicate or empty entries (which did not respond to the questionnaire). Finally, data from 388 surveys were analyzed.

### Measurements

Social background, game usage level, and health-related risk behaviors were used as measures to investigate youth health-related risk behaviors during the COVID-19 pandemic. As the participants were adolescents, parental consent was obtained because the questionnaire included negative factors such as smoking, depression, and drinking.

#### Social background

The social background of participants, including the schools they attended and their grades (middle school, high school), was collected through a survey. Students’ health-related risk behaviors vary according to their social background. The study participants were recruited on the premise that students maintain a normal relationship with adoptive parents, have no problems in school life, and do not have family discord targeting Koreans.

#### Game usage level in youth

To evaluate the severity of game usage level, this study used a modified and improved 20-item questionnaire based on the National Information Society Agency’s youth game addiction self-assessment tool and Kim’s (2017) measurement tool. Each item on the questionnaire was rated on a 4-point scale (0 = *strongly disagree*, 3 = *strongly agree*). Addiction level was categorized into as high-risk (high negative effects and no positive effects with game usage), potential-risk (high negative effects with some positive effects), and general users (no positive or negative effects). The general-user group had a total score of < 37 points, the potential-risk group had a total score between 38 and 48 points, and the high-risk group had a total score of ≥ 49 points. The Cronbach’s α of the original tool was 0.90; in this study, *α* = 0.979 and *ω* = 0.978.

#### Youth health-related risk behaviors

Data on youth health-related risk behaviors were obtained from the 2019 Korea Youth Risk Behavior Web-based Survey (KYRBS, https:/yhs.cdc.go.kr), which is conducted yearly with adolescents in middle and high school, on youth living in Seoul. The questionnaire consisted of 12 items from six categories: fatigue (1), stress (1), smoking (1), drinking (1), eating behaviors (4), and physical activity (4). The higher the total score, the higher the level of health-related risk behaviors.

### Procedure

Data were collected using an anonymous online survey between June 10 and July 21, 2020. In this study, the term “adolescents” refers to individuals aged between 15 and 24, in line with the definition used by the United Nations (UN, 2020). Therefore, all teenagers aged 16–19 living in Seoul were eligible to participate in the study. Participants in the study were recruited through teachers living in Seoul and Gyeonggi-do. Teachers and students used snowball sampling to reach more respondents.

Participants were instructed to read the consent page at the start of the online survey and click the consent button at the bottom of the page if they agreed to participate with their parents’ consent. Then, they were asked to complete an online survey. Participants were briefed on the study’s objectives, procedures and data use, and were informed that participation was entirely voluntary and that their identity would be kept confidential.

The University Ethics Committee (blinded for review) reviewed and approved the protocol of this study (approval no. blinded for review). The data collection and research procedures were performed in accordance with the Declaration of Helsinki and the policies concerning human participants, as stipulated by the authors’ institution. Written informed consent was obtained from all participants before the intervention. They were informed of their right to withdraw at any time without incurring any penalty, and were given a chance to ask for clarifications regarding the intervention.

### Data analysis

Windows SPSS 26.0 was used to analyze game participation level and health-related risk behavior data. After data cleaning and coding, the specific analytical procedures were as follows: (1) participants’ demographic characteristics were identified by descriptive and frequency analysis, (2) reliability analysis of the internal consistency between measured items was carried out using Cronbach’s ɑ, and (3) ANOVA and multivariate analysis of variance (MANOVA) were conducted to identify health-related risk behaviors based on the typical characteristics of middle-school students, and Scheffé’s method post hoc test over the data set.

## Results

### Descriptive statistics and correlation on the demographic characteristics of youth (*n* = 388)

The descriptive statistics of all dependent variables are shown in Table 2. The results were as follows: stress (median [*M*] = 3.44, standard deviation [*SD*] = 0.945), fatigue (*M* = 3.24, *SD* = 1.164), improper eating habits (*M* = 4.12, *SD* = 0.938), and physical inactivity (*M* = 4.46, *SD* = 1.535).

**Table 2:** Means, SDs, ranges

	*M* ± *SD*(range)
1. Drinking	1.69 ± 0.462(1–2)
2. Smoking	1.91 ± 0.283(1–2)
3. Stress	3.44 ± 0.945(1–5)
4. Fatigue	3.24 ± 1.164(1–5)
5. Depression	1.21 ± 0.326(1–2)
6. Improper Eating Habits	4.12 ± 0.938(1.25-6)
7. Physical Inactivity	4.46 ± 1.535(1-6.5)

A one-way ANOVA was performed to compare participants by sex in the secondary-school group for health-related risk behaviors (Table 3). As a result of the test, males showed a high level of stress (*M* = 2.76, *SD* = 0.98) (*p* < .01), and female students showed a high level of physical activity (*M* = 4.69, *SD* = 1.31) (*p* < .001).

**Table 3:** ANOVA on adolescent game usage level and resultant health-related risk behaviors by sex

Dependent variables	ANOVA		
	Sex		
	Male	Female	*f*
1. Drinking	0.66 ± 0.47	0.73 ± 0.44	2.385
2. Smoking	0.89 ± 0.32	0.94 ± 0.23	3.843
3. Stress	2.76 ± 0.98	2.31 ± 0.83	23.226***
4. Fatigue	3.15 ± 1.14	3.35 ± 1.185	2.864
5. Depression	1.28 ± 0.45	1.36 ± 0.48	2.836
6. Improper Eating Habits	4.10 ± 0.93	4.15 ± 0.95	0.261
7. Physical Inactivity	4.27 ± 1.68	4.69 ± 1.31	7.252**
8. Game Usage level	7.93 ± 3.83	7.44 ± 3.76	1.576

***p<.001, **p<.01, *p<.05

A one-way ANOVA was performed to compare participants in a group of secondary-school students for health-related risk behaviors (Table 4). As a result of the test, middle-school students’ drinking (*M* = 0.87, *SD* = 0.34), smoking (*M* = 0.99, *SD* = 0.12), depression (*M* = 3.45, *SD* = 1.07), bad eating habits (*M* = 4.30, *SD* = 0.95), lack of physical activity (*M* = 4.81, SD = 1.56), and game immersion (*M* = 9.34, *SD* = 4.06) (*p* < .001) were higher than those of high-school students. Additionally, the level of health risk behavior of middle-school students was higher than that of high-school students, and high-school students had a higher stress level than middle-school students (*M* = 1.43, *SD* = 0.50) (*p* < .001).

**Table 4:** ANOVA on adolescent game usage level and resultant health-related risk behaviors by school level

Dependent variables	ANOVA		
	School level		
	Middle School	High School	*F*
1. Drinking	0.87 ± 0.34	0.49 ± 0.50	74.035***
2. Smoking	0.99 ± 0.12	0.83 ± 0.38	32.409***
3. Stress	2.55 ± 0.97	2.57 ± 0.92	0.063
4. Fatigue	3.45 ± 1.07	2.99 ± 1.22	15.131***
5. Depression	1.22 ± 0.41	1.43 ± 0.50	20.990***
6. Improper Eating Habits	4.30 ± 0.95	3.91 ± 0.88	17.304***
7. Physical Inactivity	4.81 ± 1.56	4.06 ± 1.40	24.442***
8. Game Usage level	9.34 ± 4.06	5.82 ± 2.34	105.001***

***p<.001, **p<.01, *p<.05

### ANOVA on level of adolescent game usage (*n* = 388)

A one-way ANOVA was conducted to compare the three groups of participants on health-related risk behavior (Table 5). Results indicated that the high-risk group showed higher levels of health-related risk behavior than the general-user group. Specifically, the results are as follows: drinking (*M* = 1.89, *SD* = 0.32), smoking (*M* = 1.98, *SD* = 0.16), stress (*M* = 3.89, *SD* = 0.66), fatigue (*M* = 3.84, *SD* = 0.68), improper eating habits (*M* = 4.68, *SD* = 0.73), physical inactivity (*M* = 5.49, *SD* = 1.36) (*p* < .001). However, the general-user group showed a higher result in depressive (*M* = 1.27, *SD* = 0.35, *p* < .001) symptoms. The post hoc test revealed that the high-risk group showed significantly higher levels of health risk-related behaviors (drinking, smoking, stress, fatigue, inappropriate eating habits, lack of physical activity) than the potential-risk group and general users, and the high-risk group was more depressed than the potential-risk group.

**Table 5:** ANOVA on adolescent game usage level and resultant health-related risk behaviors

Dependent variables	ANOVA				
	General-User Group^a^	Potential-Risk Group^b^	High-Risk Group^c^	*F*	Post hoc
1. Drinking	1.58 ± 0.49	1.68 ± 0.47	1.89 ± 0.32	39.350***	c > a,b
2. Smoking	1.88 ± 0.32	1.88 ± 0.32	1.98 ± 0.16	11.865***	c > a,b
3. Stress	3.16 ± 1.01	3.50 ± 0.85	3.89 ± 0.66	53.871***	c > a,b
4. Fatigue	2.92 ± 1.22	3.10 ± 1.28	3.84 ± 0.68	65.061***	c > a,b
5. Depression	1.27 ± 0.35	1.23 ± 0.31	1.12 ± 0.27	17.804***	a > c, b > c
6. Improper Eating Habits	3.84 ± 0.92	3.95 ± 0.89	4.68 ± 0.73	83.571***	c > a,b
7. Physical Inactivity	3.93 ± 1.39	4.18 ± 1.31	5.49 ± 1.36	102.006***	c > a,b

***p<.001, **p<.01, *p<.05

### MANOVA analysis on adolescent game usage level (*n* = 388)

A MANOVA was conducted to compare the three groups’ health-related risk behaviors (Table 6). Results indicated that female students in the high-risk group showed higher stress levels (*f* = 5.835, *p* < .01, Cohen’s *d* = 0.017) and fatigue (*f* = 5.549, *p* < .01, Cohen’s *d* = 0.016) than males of the same group. However, male students showed higher physical inactivity levels (*f* = 3.195, *p* > .05, Cohen’s *d* = 0.009) than females. Depression levels were higher among the female students in the general-user group, but this difference was non-significant. The results on sex differences among the three groups were Wilks’ *λ* = 0.947, *F* (14) = 2.676, *p* = .001, and $\eta_p^2$ *=* 0.027. The post hoc test indicated clear sex distinctions among the general, potential, and high-risk groups on excessive gaming (*p* < .001).

**Table 6:** MANOVA on level of gaming use and corresponding health-related risk behaviors by sex

Dependent variables	MANOVA					
	Sex (Male vs. Female)					
	General-User Group	Potential-Risk Group	High-Risk Group	*f*	Eta^2^	
1. Drinking	1.51 ± 0.50 vs. 1.66 ± 0.48	1.71 ± 0.46 vs. 1.65 ± 0.48	1.87 ± 0.34 vs. 1.90 ± 0.30	2.719	0.008	
2. Smoking	1.84 ± 0.37 vs. 1.93 ± 0.26	1.88 ± 0.33 vs. 1.88 ± 0.32	1.96 ± 0.20 vs. 2.00 ± 0.00	1.132	0.003	
3. Stress	2.84 ± 0.99 vs. 3.51 ± 0.92	3.32 ± 0.87 vs. 3.73 ± 0.77	3.80 ± 0.71 vs. 4.00 ± 0.56	5.835**	0.017	
4. Fatigue	2.81 ± 1.23 vs. 3.04 ± 1.20	2.79 ± 1.11 vs. 3.50 ± 1.38	3.83 ± 0.56 vs. 3.85 ± 0.82	5.549**	0.016	
5. Depression	1.21 ± 0.34 vs. 1.32 ± 0.37	1.22 ± 0.25 vs. 1.23 ± 0.37	1.10 ± 0.20 vs. 1.14 ± 0.32	1.377	0.004	
6. Improper Eating Habits	3.79 ± 0.90 vs. 3.90 ± 0.93	3.86 ± 0.95 vs. 4.06 ± 0.81	4.69 ± 0.64 vs. 4.67 ± 0.84	0.869	0.003	
7. Physical Inactivity	3.59 ± 1.47 vs. 4.32 ± 1.19	4.06 ± 1.39 vs. 4.35 ± 1.18	5.44 ± 1.48 vs. 5.57 ± 1.17	3.195*	0.009	
Wilks’ λ	F	Hypothesis Degree of Freedom	Error Degree of Freedom	Partial η^2^	Noncentrality Parameter	Observed Power^d^
0.947	2.676b	14.000	1360.000	0.027	37.463	0.992

Fatigue (*f* = 11.584, *p* < .001, Cohen’s *d* = 0.033), improper eating habits (*f* = 3.185, *p* < .01, Cohen’s *d* = 0.014), and physical inactivity (*f* = 20.405, *p* < .001, Cohen’s *d* = 0.032) levels were higher among middle-school students than high-school students. However, depression (*f* = 4.893, *p* < .01, Cohen’s *d* = 0.014) levels were higher among high-school students than middle-school students. The results by school level (middle or high school) among the three groups were Wilks’ *λ* = 0.901, *F* (14) = 5.208, *p* = .000, and $\eta_p^2$ *=* 0.051. The post hoc test indicated there were clear distinctions by school level among the general, potential, and high-risk groups on excessive gaming (*p* < .001) (Table 7).

**Table 7:** MANOVA on level of gaming use and corresponding health-related risk behaviors by school level

Dependent variables	MANOVA					
	School (Middle School vs. High School)					
	General-User Group	Potential-Risk Group	High-Risk Group	*f*	Eta^2^	
1. Drinking	1.81 ± 0.39 vs. 1.45 ± 0.50	1.81 ± 0.39 vs. 1.58 ± 0.50	1.92 ± 0.28 vs. 1.69 ± 0.47	1.630	0.005	
2. Smoking	1.97 ± 0.16 vs. 1.83 ± 0.38	2.00 ± 0.00 vs. 1.79 ± 0.41	1.99 ± 0.097 vs. 1.87 ± 0.33	1.456	0.004	
3. Stress	2.91 ± 1.09 vs. 3.31 ± 0.94	3.33 ± 0.91 vs. 3.64 ± 0.78	3.87 ± 0.65 vs. 4.00 ± 0.71	1.242	0.004	
4. Fatigue	3.01 ± 1.26 vs. 2.87 ± 1.20	2.81 ± 1.26 vs. 3.33 ± 1.26	3.92 ± 0.53 vs. 3.31 ± 1.17	11.584***	0.033	
5. Depression	1.22 ± 0.33 vs. 1.29 ± 0.37	1.17 ± 0.27 vs. 1.27 ± 0.33	1.08 ± 0.22 vs. 1.34 ± 0.34	4.893**	0.014	
6. Improper Eating Habits	3.82 ± 0.99 vs. 3.85 ± 0.87	3.92 ± 0.93 vs. 3.97 ± 0.87	4.74 ± 0.69 vs. 4.30 ± 0.83	3.185**	0.014	
7. Physical Inactivity	3.94 ± 1.30 vs. 3.93 ± 1.44	4.00 ± 1.39 vs. 4.33 ± 1.22	5.63 ± 1.31 vs. 4.56 ± 1.30	20.405***	0.032	
Wilks’ λ	F	Hypothesis Degree of Freedom	Error Degree of Freedom	Partial η^2^	Noncentrality Parameter	Observed Power^d^
0.901	5.208b	14.000	1360.000	0.051	72.917	1.000

Finally, participants who engaged in mobile gaming had higher levels of physical inactivity (*f* = 13.801, *p* < .001, Cohen’s *d* = 0.039) than those who engaged in online gaming. However, smoking (*f* = 5.294, *p* > .01, Cohen’s *d* = 0.015) was higher in both online and mobile gaming groups among high-risk users than the potential and general users. There were significant differences in various health-related risk behaviors among the gaming groups. Specifically, the post hoc test indicated that participants who engaged in gaming also engaged in more health-related risk behaviors; however, it was shown that the general-user group engaged in more health-related risk behaviors. The results on differences in type of games among the three groups were Wilks’ *λ* = 0.937, *F* (14) = 3.200, *p* = .000, and $\eta_p^2$ *=* 0.032. The post hoc test indicated there were clear distinctions in mobile and online gaming among the three groups for excessive gaming (*p* < .001) (Table 8).

**Table 8:** MANOVA on level of gaming use and corresponding health-related risk behaviors by game type

Dependent variables	MANOVA					
	Type of Games (Online Game vs. Mobile Game)					
	General-User Group	Potential-Risk Group	High-Risk Group	*f*	Eta^2^	
1. Drinking	1.49 ± 0.50 vs. 1.66 ± 0.48	1.64 ± 0.48 vs. 1.75 ± 0.44	1.88 ± 0.33 vs. 1.90 ± 0.30	2.046	0.006	
2. Smoking	1.82 ± 0.39 vs. 1.94 ± 0.23	1.83 ± 0.38 vs. 1.96 ± 0.20	1.98 ± 0.16 vs. 1.98 ± 0.16	5.294**	0.015	
3. Stress	2.97 ± 1.07 vs. 3.34 ± 0.92	3.39 ± 0.86 vs. 3.67 ± 0.81	3.85 ± 0.63 vs. 3.95 ± 0.70	1.850	0.005	
4. Fatigue	2.90 ± 1.21 vs. 2.94 ± 1.23	3.08 ± 1.33 vs. 3.13 ± 1.21	3.84 ± 0.53 vs. 3.83 ± 0.91	0.061	0.000	
5. Depression	1.25 ± 0.35 vs. 1.28 ± 0.36	1.21 ± 0.28 vs. 1.25 ± 0.36	1.09 ± 0.19 vs. 1.18 ± 0.35	0.951	0.003	
6. Improper Eating Habits	3.84 ± 0.93 vs. 3.85 ± 0.91	3.95 ± 0.90 vs. 3.94 ± 0.89	4.67 ± 0.74 vs. 4.71 ± 0.70	0.049	0.000	
7. Physical Inactivity	3.44 ± 1.41 vs. 4.39 ± 1.20	4.15 ± 1.43 vs. 4.23 ± 1.12	5.59 ± 1.38 vs. 5.30 ± 1.29	13.801***	0.039	
Wilks’ λ	F	Hypothesis Degree of Freedom	Error Degree of Freedom	Partial η^2^	Noncentrality Parameter	Observed Power^d^
0.937	3.200b	14.000	1360.000	0.032	44.794	0.998

## Discussion

Owing to the COVID-19 lockdown measures and consequent lack of social interactions at school, the surge in adolescent smartphone addiction and excessive gaming is a growing concern that can lead to youth engaging in negative behaviors such as drinking or smoking. However, decreased non-gaming leisure activities and subsequent excessive gaming behavior influenced by social distancing policies and increased health-related risk behaviors merit further research. Thus, this study aimed to discern the differences in health-related risk behavior factors brought on by the COVID-19 social distancing protocols.

This study classified health-related risk behavior such as drinking, smoking, stress, depression, improper eating habits, and physical inactivity, and the results showed a meaningful correlation with increased gaming levels. In other words, these behaviors can be considered social isolation-related stress-induced adolescent health-related risk behaviors. A recent study by the Korea Creative Content Agency (KOCCA, 2022) showed that young adults gamed more, performed worse in school, and were more depressed than before the COVID-19 pandemic. Thus, this study determined the health-related risk behavior differences by sex, school level, and type of gaming (online or mobile).

Students classified as high-risk gamers showed higher levels of health-related risk behaviors than those who were general users. Similar studies have found that physical inactivity coupled with poor health behaviors can bring about adverse health outcomes at a young age (Bang, 2010; Kim, 2018; Puolitaival et al., 2020). Both male and female high-risk students showed higher levels of health-related risk behaviors than the general-user group; however, female students had overall higher levels of risk behaviors than their male counterparts. Numerous studies have asserted that male students are more likely than females to become excessive gamers (Colder Carras et al., 2017; Gentile, 2009), and studies on game usage and leisure activity during the COVID-19 school closures support this notion (Cho, 2022; Zhu et al., 2021). Yet, the results of this study showed that female students in the high-risk group had higher levels of risk behaviors than male students.

Generally, it has been thought that males are more impulsive (Ahrends, 2017), which affects autonomy and skills when it comes to gaming (Dursun & Capan, 2018; Reinecke et al., 2012). However, Lopez-Fernandez et al. (2019) argued that game addiction research has been focused on male gamers, although there is a steady increase in female gamers (Savci et al., 2021). Hence, looking at the present results through previous literature indicates there is an increase in female participation in gaming, as well as an increase in game usage. Therefore, it can be assumed that COVID-19 social distancing caused an increase in female student game usage, and, consequently, an increase in excessive gaming.

A study by Rodríguez-Fernández et al. (2021) found that women—especially younger women—were prone to display negative feelings such as depression, anxiety, stress, apprehension, PTSD, and rage due to social isolation brought on by the COVID-19 pandemic. Additionally, Zhu et al. (2021) asserted that female young adults who exhibited signs of pathological and extreme game addiction were more prone to feel loneliness. In this study, female middle-school students were especially susceptible to engaging in health-related risk behaviors. As adolescent females’ dependency on intimacy with others and peer relationships increased due to social isolation, it is likely that their level of loneliness and worries over losing relationships increased as well (Henrich et al., 2001; Rose & Rudolph, 2006). In other words, it is highly likely that female young adults engage in online gaming to form and maintain social interactions in response to negative feelings brought on by social isolation (Di Blasi et al., 2019; Kardefelt-Winther, 2014; Király & Potenza, 2020). Therefore, COVID-19 and social isolation are more likely to have more negative impacts on men than on women, and game usage is a high contributing factor.

Online gaming impacts young adults mentally and physically (Zhang et al., 2021). Lack of outdoor activity increases the time spent watching TV or gaming, and induces improper eating habits. Sedentary lifestyles brought on by the COVID-19 pandemic have led to teenage obesity (Park, 2020; Łuszczki et al., 2021). A study by Łuszczki et al. (2021) indicated that the proportion of overweight young adults increased from 11.2 to 13.4%, but that low body weight had also increased from 11.7 to 15.0%. Cho (2019) found that female youth were more sedentary than males and more prone to depression and thoughts of suicide. This finding aligned with Yeom’s (2018) finding that women were more likely to smoke. Further, women were also more likely to have improper eating habits.

While game usage severity did not vary between the school levels in this study, the risk behavior factors varied between middle- and high-school students. Middle-school students showed higher levels of fatigue, improper eating habits, and physical inactivity, whereas high-school students had higher levels of depression. A study by Zhu et al. (2021) asserted that game usage increased among youth during the pandemic, but gaming helped alleviate stress to a degree. Despite these findings, youth engaging in excessive gaming produces negative health impacts.

While it has been shown that excessive gaming brought about a more sedentary lifestyle and caused young adults to be more tired and engage in improper eating habits, it is necessary to investigate why high-school students showed higher depression levels with increased gaming. A study on the impact of adolescent smartphone addiction indicated that the greater the addiction, the higher students’ levels of depression and anxiety (Jung, 2014; Lee, 2014). Additionally, according to the Adolescent Game Addiction Survey (KOCCA, 2022), adolescent gaming behavior was closely tied to their emotional state. This study found that young adults who engaged in excessive gaming behavior displayed more prominent negative characteristics than the other groups; they showed low levels of life satisfaction and self-esteem while also responding that they received less care and attention from their parents. The high-risk group displayed higher levels of anxiety, depression, impulsiveness, carelessness, and school-related stress than the other groups. It has been shown that even though school learning decreased due to COVID-19 closures, the burden of studying increased via private academies and other extracurricular learning activities, leading to stress and low quality of life (Ki & Cho, 2021). Research conducted by Rajab et al. (2020) claimed that stress had similar correlations as game addiction and can have negative impacts on familial and societal relationships, as well as on academic achievement, self-control, and restraint. It has also been cited that depression levels increase as gaming addiction and immersion increase (Seo, 2011). Therefore, it can be assumed that gaming to relieve stress can bring about more stress related to excessive gaming, which, in turn, leads to more health-related risk behaviors.

Lastly, this study also identified the differences in risk behaviors as more young adults turned to mobile gaming due to COVID-19 closures of leisure activity places such as game cafes and playrooms. Both mobile and online high-risk users displayed a significantly high level of adolescent smoking compared to the other groups. A study on the connection of adolescent experiences with smoking and internet addiction confirmed that excessive adolescent internet and computer usage was closely tied with excessive drinking and smoking in early adulthood (Kim et al., 2012).

The study results indicated that students who played mobile games showed higher levels of health-related risk behaviors. This finding could be because more than 80% of the population has access to a smartphone, making it easy to access mobile games anywhere at any time. As more health-related risk behavior with heavy game usage leads to excessive gaming and more risk behaviors, policies on adolescent game usage are necessary. Above all, the aforementioned results all suggest a correlation between adolescent game usage behavior and parental attitude, illustrating the need for parental support for children’s gaming habits.

The findings of the present study provide insight that may be useful for policymakers and mental health professionals in considering and developing appropriate interventions to treat adolescent online gaming addiction and the consequent negative physical and mental impacts. Parents need to adopt a supportive attitude instead of forcibly restraining their children from gaming. In other words, parents should support and teach their children how to develop self-restraint and to perceive gaming as a positive way of spending free time. Several studies have reported that parents play a vital role in preventing adolescent game addiction (Donati et al., 2021; Elsayed, 2021; Király et al., 2020). Parents must appropriately guide their children by recognizing their gaming patterns and monitoring and helping them control their gaming time since their knowledge of their children is a protective factor against social and psychological issues (Elsayed, 2021).

In addition, Han et al. (2022) reported that the psychological impact of COVID-19 on children and youth game addiction has been studied and that parents play an important role in preventing negative effects. They also suggested that intensive interventions should be designed for emotionally vulnerable children and adolescents. Therefore, in addition to providing parental guidance and support, gaming addiction should be considered an emotional and behavioral disorder requiring counseling experts and professionals to join forces with parents to provide a cure and reform program (Elsayed, 2021; Király et al., 2020; Oliveira et al., 2022; Viana et al., 2021). Thus, adolescent gaming addiction should not simply be regarded as the individual’s problem; instead, it is a problem that requires the protection of the mental health safety net system as well as institutional and policy reforms of the educational system and the community to ensure adolescents’ health and well-being. In this vein, the Korea Youth Counseling and Welfare Institute operates counseling centers in every region, providing online and in-person counseling services, educational programs to parents, counseling camps for excessive gaming, and support for expenses (Jung & Song, 2022; Jhone & Lee, 2022).

### Limitations

Due to the scoring method, the game usage level scale used in this survey has limitations in the interpretation by type. As it is difficult to precisely compare the intensity of problematic and normal game use between types, supplementing the scale is important. To this end, it is necessary to develop a scale that can distinguish between groups with potential problems and those with very serious problems and determine whether to use problematic games. In this regard, a diagnostic tool must be developed to comprehensively measure youth game literacy, game use problems, and satisfaction through games. Such a tool would be able to grasp the status of game use and overindulgence among adolescents more accurately. Survey results obtained using this proposed scale could generate useful data for establishing policies to foster a healthy gaming culture for children and adolescents in Korea.

In addition, convenience sampling, a non-probability sampling technique, was used in this study. Typically, a representative sample is needed; however, this technique may be more appropriate when probability sampling is not possible, is expensive, time-consuming, and impractical. Therefore, future research should be conducted on game fairs where a representative sample of students receiving treatment for game addiction can be obtained.

Lastly, in the survey procedure of this study, instructions were given to proceed with the survey after obtaining parental consent, but it is possible that some students participated without parental/guardian consent, because parental/guardian consent was not cross-checked. However, as the survey was anonymous, the risk to respondents was low. For this part, it will be necessary to proceed with the survey in the presence of parents in future research.

## Conclusion

This study chose middle- and high-school students as participants to investigate the effects of game usage on health-related risk behaviors. Participants were divided into groups according to school level, game usage level, and whether they played online or mobile games to examine the correlation between the risk behavior factors.

First, students who showed high levels of game usage also showed high levels of health-related risk behaviors, with significant sex-related differences in the high-risk group in risk behavior. In previous studies it was generally thought that male students showed gaming addiction behaviors, however, this study found that female students had higher risk behavior levels than the male students in the high-risk group. The levels were especially high in stress, physical inactivity, depression, smoking, and fatigue.

Second, middle-school students exhibited higher levels of risk behavior than high-school students. This shows that while high-school students have less leisure activity time due to college entrance preparations, middle-school students have more leisure time but have higher levels of stress due to social isolation brought on by COVID-19 restrictions. Accordingly, middle-school students appeared to have higher levels of health-related risk behaviors than their high-school counterparts.

Third, the study categorized the participants by their preferred platform and compared risk behaviors among the mobile and online high-risk user groups. Both online and mobile gaming high-risk groups had higher levels of smoking behavior than the general-user groups, with mobile gamers engaging in riskier behaviors than online gamers. This can be attributed to the fact that mobile games are more readily accessible and have fewer limitations than online gaming, highlighting the need for policies on adolescent mobile game usage.

## Data Availability

The original contributions presented in the study are included in the article/Supplementary material, further inquiries can be directed to the corresponding author.
